# Survival Outcomes in Patients With Hormone Receptor–Positive Metastatic Breast Cancer With Low or No *ERBB2* Expression Treated With Targeted Therapies Plus Endocrine Therapy

**DOI:** 10.1001/jamanetworkopen.2023.13017

**Published:** 2023-05-11

**Authors:** Jason A. Mouabbi, Akshara Singareeka Raghavendra, Roland L. Bassett, Amy Hassan, Debasish Tripathy, Rachel M. Layman

**Affiliations:** 1 Department of Breast Medical Oncology, The University of Texas MD Anderson Cancer Center, Houston; 2 Department of Biostatistics, The University of Texas MD Anderson Cancer Center, Houston; 3 Department of General Oncology, The University of Texas MD Anderson Cancer, Houston

## Abstract

**Importance:**

Approximately 45% to 60% of hormone receptor (HR)–positive metastatic breast cancer (mBC) shows a low-level expression of *ERBB2*. Low *ERBB2* expression is defined as *ERBB2* immunohistochemical expression of 1+ or 2+ with a negative *ERBB2* amplification by in situ hybridization. The efficacy of the antibody-drug conjugate trastuzumab deruxtecan in low-*ERBB2*, HR-positive mBC has been practice changing. However, there are conflicting data on the prognostic value of low *ERBB2* expression in HR-positive mBC and whether low *ERBB2* expression is a separate entity.

**Objective:**

To examine whether outcomes differ by immunohistochemical analysis for patients with HR-positive mBC with low *ERBB2* expression vs those without *ERBB2* expression when treated with targeted therapy (TT) plus endocrine therapy (ET).

**Design, Setting, and Participants:**

This single-institution cohort study used prospectively collected electronic data from the MD Anderson Cancer Center for patients with a diagnosis of HR-positive mBC treated with ET in combination with a TT (cyclin-dependent kinase 4/6 inhibitors [CDK4/6is], everolimus, or alpelisib) between January 1, 2010, and December 31, 2021.

**Exposure:**

HR-positive mBC with either low or no *ERBB2* expression.

**Main Outcome and Measures:**

The main outcomes were median progression-free survival and overall survival. Data on demographic characteristics, estrogen and progesterone receptor status, *ERBB2* status, histologic subtype, menopausal status, treatment duration, and survival status were collected.

**Results:**

A total of 1585 women (median [range] age, 51 [24-92] years) were included in the study. Of these women, 1013 (63.9%) had mBC with low *ERBB2* expression and 572 (36.1%) had mBC with no *ERBB2* expression. A total of 1084 (68.4%) were treated with a CDK4/6i (912 patients were treated in the first line and 172 were treated in the second line); 475 (30.0%) received everolimus and 26 (1.6%) received alpelisib. In the patients who received a first-line CDK4/6i, 618 (67.8%) received an aromatase inhibitor as their ET backbone and 265 (29.1%) received fulvestrant. With a median follow-up time of 17.9 months (range, 1-111 months), progression-free survival and overall survival were not statistically different between the patients with low and no *ERBB2* expression treated with TT plus ET.

**Conclusions and Relevance:**

In this cohort study of patients with HR-positive mBC treated with TT plus ET, low *ERBB2* expression did not have a significant association with prognosis.

## Key Points

**Question:**

Do outcomes differ for patients with hormone receptor (HR)–positive metastatic breast cancer (mBC) with low *ERBB2* expression compared with those with no *ERBB2* expression when treated with targeted therapies in combination to endocrine therapy (ET)?

**Findings:**

In this cohort study of 1585 patients, the outcomes for patients with HR-positive mBC with low *ERBB2* expression were similar to those for patients with no *ERBB2* expression when treated with targeted therapies in combination with ET. This concordance applied similarly to both ductal and lobular histologic subtypes.

**Meaning:**

Low *ERBB2* status did not have a significant association with prognosis in patients with HR-positive mBC treated with targeted therapies plus ET.

## Teaser

This cohort study of women with hormone receptor–positive metastatic breast cancer treated with targeted therapy in combination with endocrine therapy compares the outcomes for those with low vs no *ERBB2* expression.

## Introduction

Hormone receptor (HR)–positive, *ERBB2* (formerly *HER2* or *HER2*/*neu*)–negative (OMIM 164870) metastatic breast cancer (mBC) is the most common subtype of mBCs, seen in approximately 60% of patients.^1^ Historically, these tumors were treated with endocrine therapy (ET).^2^ However, the development of targeted therapies (TTs), such as the cyclin-dependent kinase 4/6 inhibitors (CDK4/6is), the mammalian target of rapamycin inhibitor everolimus, and the phosphoinositide 3-kinase inhibitor alpelisib, and their combination with ET have transformed the management of such tumors.^1^ Until recently, patients with HR-positive, *ERBB2*-negative breast cancer have been treated with ET and TT until their disease becomes hormone refractory, and at that point, they start sequential single-agent chemotherapies.^1^ The 2022 American Society of Clinical Oncology guidelines have changed this paradigm with the introduction of 2 antibody-drug conjugates to the field of HR-positive mBC: trastuzumab deruxtecan^3,4^ and sacituzumab govitecan.^5,6^

The DESTINY-Breast04 (Trastuzumab Deruxtecan [DS-8201a] vs Investigator's Choice for HER2-Low Breast Cancer That Has Spread or Cannot Be Surgically Removed) trial showed that when treated with trastuzumab deruxtecan, patients with mBC that expresses low levels of *ERBB2* had significantly improved outcomes compared with standard-of-care therapies.^3^ Low *ERBB2* expression is defined as *ERBB2* immunohistochemical expression of 1+ or 2+ with a negative *ERBB2* amplification by in situ hybridization.^7^ The impressive results of this study led to the US Food and Drug Administration approving trastuzumab deruxtecan for use in patients with HR-positive breast cancer with low *ERBB2* expression who have received prior ET and at least 1 prior chemotherapy in the metastatic setting.^8^ This approval swiftly created a potentially new important subtype of breast cancer, given that 45% to 60% of HR-positive mBCs have low *ERBB2* expression.^9,10^

Low *ERBB2* expression has been poorly characterized as an entity, and its impact on prognosis has been controversial. A previous study^11^ supports it as a prognostic marker for better outcomes, another^12^ shows that it is a prognostic marker for worse outcomes, and still another^13^ shows that it has no effect on prognosis. Two previous studies have found that low *ERBB2* expression is associated with a poor prognosis in patients with HR-positive mBC treated with CDK4/6is when compared with those who have no *ERBB2* expression by immunohistochemical analysis.^12,14^ Two other contemporaneous studies showed that low *ERBB2* expression did not affect the response to CDK4/6is.^15,16^ More data are needed to better understand whether low *ERBB2* expression is truly prognostic and whether it merits consideration as a distinct entity, especially in HR-positive mBC, given the contradicting information. In addition, it is important to determine whether the implications of low *ERBB2* expression are similar for different histologic subtypes (ie, ductal vs lobular).

In this study, we explore whether the outcomes of patients with HR-positive mBC with low *ERBB2* expression treated with TT (CDK4/6is, everolimus, or alpelisib) in combination with ET are different than the outcomes for those without *ERBB2* expression. We also explore whether those outcomes are different in invasive lobular carcinoma (ILC) and invasive ducal carcinoma (IDC).

## Methods

### Study Population and Variables

For this cohort study, we searched the institutional review board–approved breast cancer database (prospectively collected data) at The University of Texas MD Anderson Cancer Center to identify patients with HR-positive mBC with low *ERBB2* expression or no *ERBB2* expression who had been treated with ET in combination with TT (CDK4/6i, everolimus, or alpelisib) between January 1, 2010, and December 31, 2021. Participant race and ethnicity were reported to document fair and similar representation of each subpopulation in the different groups being analyzed. This study was granted a patient consent waiver by The University of Texas MD Anderson Cancer Center Institutional Review Board because it posed minimal risk and obtaining consent from patients no longer in follow-up was impractical. We followed the Strengthening the Reporting of Observational Studies in Epidemiology (STROBE) reporting guideline.

Data on patient demographic characteristics, estrogen and progesterone receptor status, *ERBB2* status (assessed at the time of metastatic diagnosis), ductal vs lobular histologic subtype, menopausal status, treatment received, treatment duration, survival, and last follow-up were collected. Patients with HR-positive mBC with low *ERBB2* expression and no *ERBB2* expression who received TT plus ET were analyzed in 3 groups: all histologic subtypes, ILC, and IDC. Progression-free survival (PFS) and overall survival (OS) duration data generated from all treatment categories were compared among all 3 groups.

### Statistical Analysis

Wilcoxon rank sum tests were used to compare the distribution of continuous variables among histologic types. Fisher exact tests were used to compare the distribution of categorical variables.

The Kaplan-Meier method was used to estimate the distribution of OS duration from the date of the initiation of treatment to the time of death or last follow-up. Patients who were still alive were censored at their last contact date. Duration of PFS was defined as the time from the date of the initiation of treatment to the date of the end of treatment. Patients who had no end date were censored at the time of last contact.

Survival distributions were compared among those with low vs no *ERBB2* expression using the log-rank test. All statistical analyses were performed using R software, version 4.1.1 (R Foundation for Statistical Computing). A 1-sided *P* < .05 was considered statistically significant. No adjustments were made for multiple testing. Figures were generated using GraphPad Prism, version 9 (GraphPad Inc).

## Results

### Baseline Characteristics

**Figure 1.**
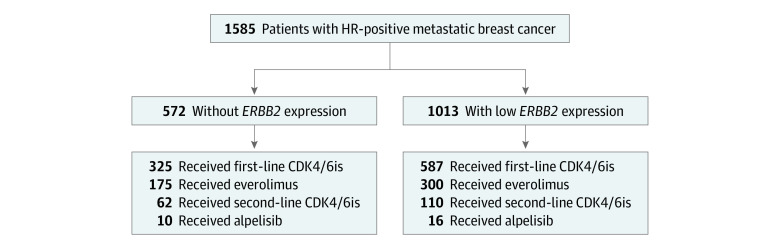
Study Cohort Flow Diagram CDK4/6is indicates cyclin-dependent kinase 4 and 6 inhibitors; HR, hormone receptor.

Between 2010 and 2021, we identified 1585 patients with HR-positive mBC. Of these women, 1013 (63.9%) had mBC with low *ERBB2* expression and 572 (36.1%) had mBC with no *ERBB2* expression (Figure 1). In the overall population, the median age was 51 years (range, 24-92 years); 140 (8.8%) identified as Black, 137 (8.6%) as Hispanic, 1186 (74.8%) as White, and 122 (7.7%) as other race or ethnicity. A total of 828 (52.2%) were premenopausal, 1342 (84.7%) had IDC, 243 (15.3%) had ILC, 1522 (96.0%) had estrogen receptor–positive tumors, and 1304 (82.3%) had progesterone receptor–positive tumors; 1084 (68.3%) received CDK4/6is, 475 (30.0%) received everolimus, and 26 (1.6%) received alpelisib. Palbociclib was the predominant CDK4/6i used (1001 cases [92.3%]) followed by abemaciclib (50 cases [4.6%]) and ribociclib (33 cases [3.0%]). Of the patients who received CDK4/6is, 912 (84.1%) received it in the first-line setting and 172 (15.9%) in the second-line setting (Table); 618 patients (67.8%) who received a first-line CDK4/6i received an aromatase inhibitor as their ET backbone and 265 (29.1%) received fulvestrant. All patients who received first-line fulvestrant had disease recurrence while taking or shortly after taking an adjuvant aromatase inhibitor. There was no statistically significant difference in clinicopathologic features and treatments received between the low *ERBB2* and no *ERBB2* expression groups (Table; eTables 1 and 2 in Supplement 1).

**Table.** Demographic and Clinicopathologic Characteristics of the Patient Cohort^a^

Characteristic	No *ERBB2* expression (n = 572)	Low *ERBB2* expression (n = 1013)	Unadjusted *P* value
Age, median (range), y	50 (24-92)	51 (20-87)	.78
Race			
Black	41 (7.2)	99 (9.7)	.13
Hispanic	57 (9.9)	80 (7.8)	
White	425 (74.3)	761 (75.1)	
Other^b^	49 (8.6)	73 (7.4)	
Menopausal status			
Pre	309 (54.0)	519 (51.2)	.29
Post	263 (46.0)	494 (48.8)	
Histologic subtype			
IDC	475 (83.0)	867 (85.5)	.19
ILC	97 (17.0)	146 (14.5)	
Estrogen receptor			
Positive	544 (95.1)	978 (96.5)	.18
Negative	28 (4.9)	35 (3.5)	
Progesterone receptor			
Positive	474 (82.8)	830 (81.9)	.68
Negative	98 (17.2)	183 (18.1)	
Targeted therapy			
CDK4/6i	387 (67.6)	697 (68.8)	.88
First line	325 (83.9)	587 (84.2)	
Second line	62 (16.1)	110 (15.8)	
Everolimus	175 (30.5)	300 (29.6)	
Alpelisib	10 (1.9)	16 (1.6)	

Abbreviations: CDK4/6i, cyclin-dependent kinase 4 and 6 inhibitors; IDC, invasive ductal carcinoma; ILC, invasive lobular carcinoma.

^a^ Data are presented as number (percentage) of study patients unless otherwise indicated.

^b^ Other includes Asian, American Indian, and Pacific Islander.

### Outcomes in Patients Treated With TT and ET

**Figure 2.**
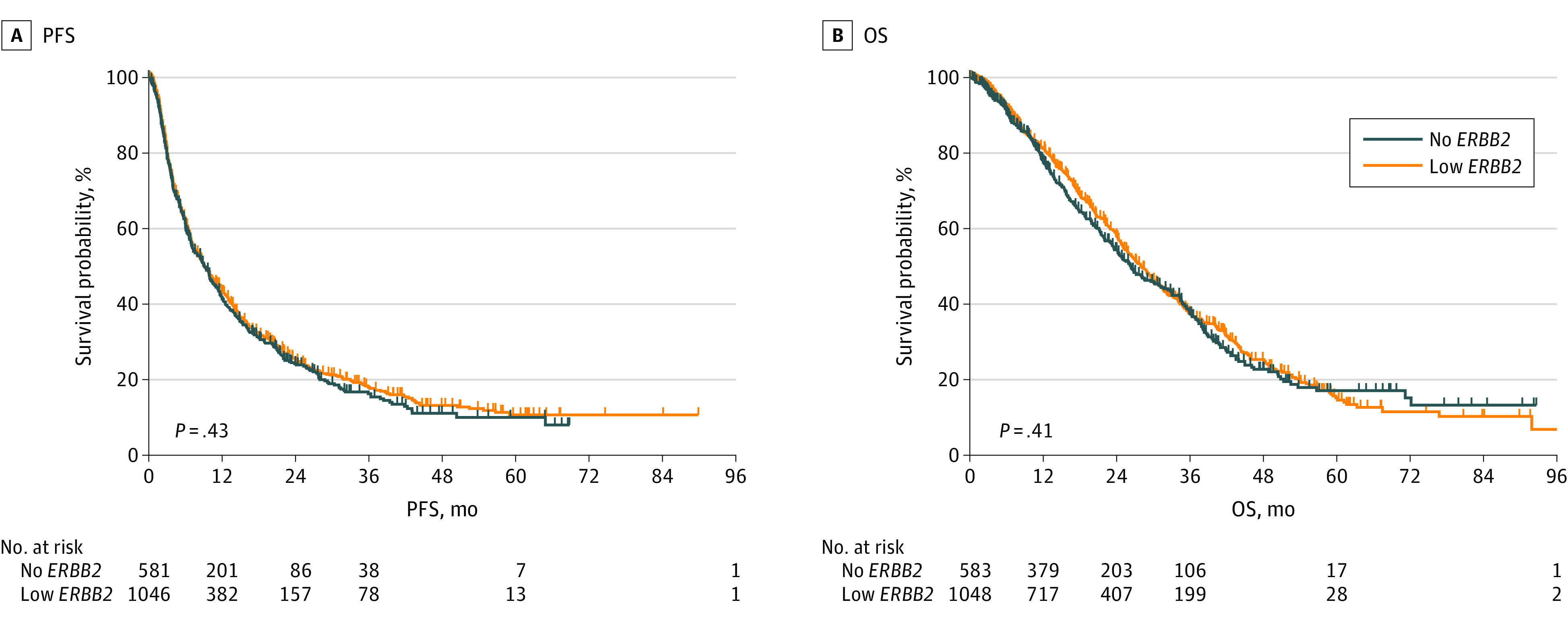
Progression-Free Survival (PFS) and Overall Survival (OS) in Patients With Metastatic Breast Cancer With Hormone Receptor–Positive Low vs No *ERBB2* Expression Treated With Targeted Therapy in Combination With Endocrine Therapy

The median follow-up time was 17.9 months with a range from 1 to 111 months. Patients with low *ERBB2* and no *ERBB2* expression treated with TT and ET had the same median PFS (mPFS) of 9.1 months (range, 0.3-89.9 months) (Figure 2A) and no statistically significant difference in median OS (mOS) (28.1 vs 26.4 months; hazard ratio [HR], 1.05; 95% CI, 0.92 - 1.21; *P* = .41) (Figure 2B). In patients with IDC, there was no statistically significant difference in mPFS (9.5 vs 8.5 months; HR, 1.12; 95% CI, 0.97-1.28; *P* = .10) (eFigure 1A in Supplement 1) and mOS (28.8 vs 26.4 months; HR, 1.07; 95% CI, 0.91-1.25; *P* = .38) (eFigure 1B in Supplement 1). In patients with ILC, there was no statistically significant difference in mPFS (9.5 vs 12.6 months; HR, 0.83; 95% CI, 0.61-1.14; *P* = .26) (eFigure 2A in Supplement 1) and mOS (27.9 vs 34.3 months; HR, 1.04; 95% CI, 0.73-1.48; *P* = .79) (eFigure 2B in Supplement 1).

### Outcomes in Patients Treated With First-line CDK4/6is and ET

**Figure 3.**
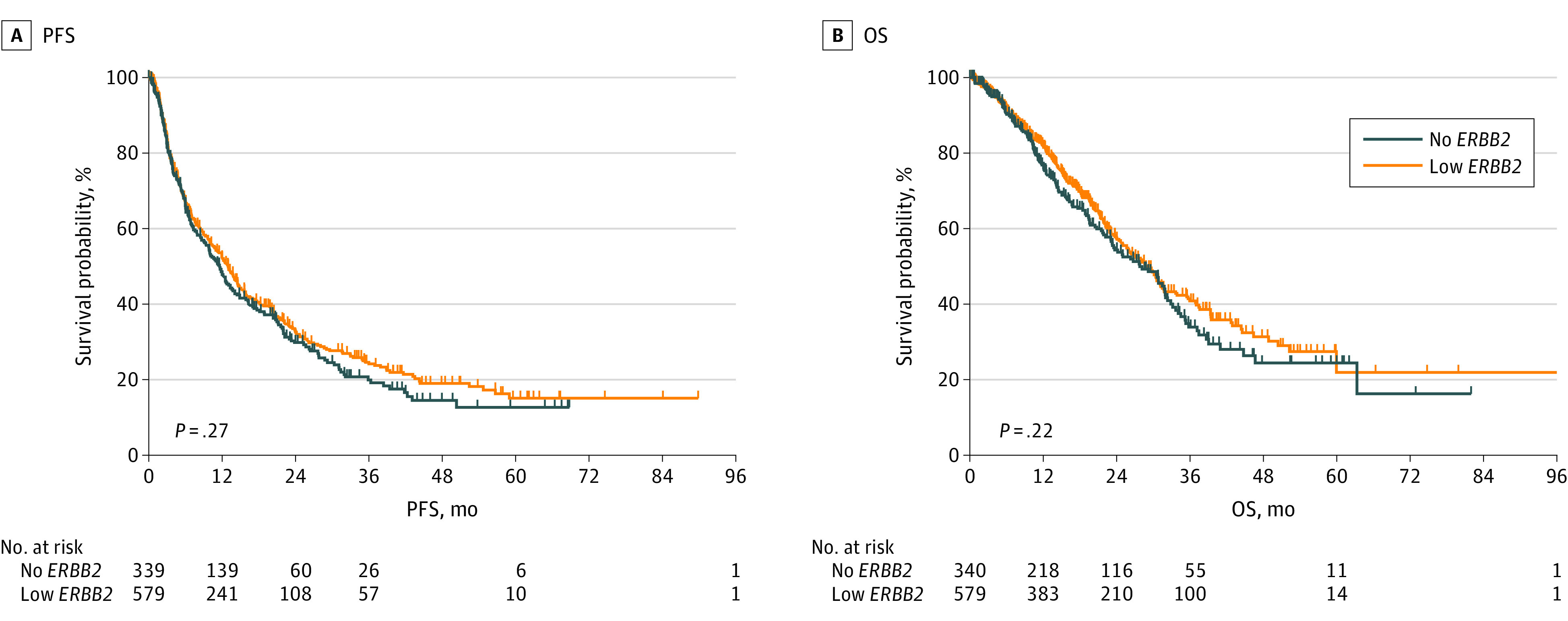
Progression-Free Survival (PFS) and Overall Survival (OS) in Patients With Metastatic Breast Cancer With Hormone Receptor–Positive Low vs No *ERBB2* Expression Treated With First-line Cyclin-Dependent Kinase 4 and 6 Inhibitors in Combination With Endocrine Therapy

**Figure 4.**
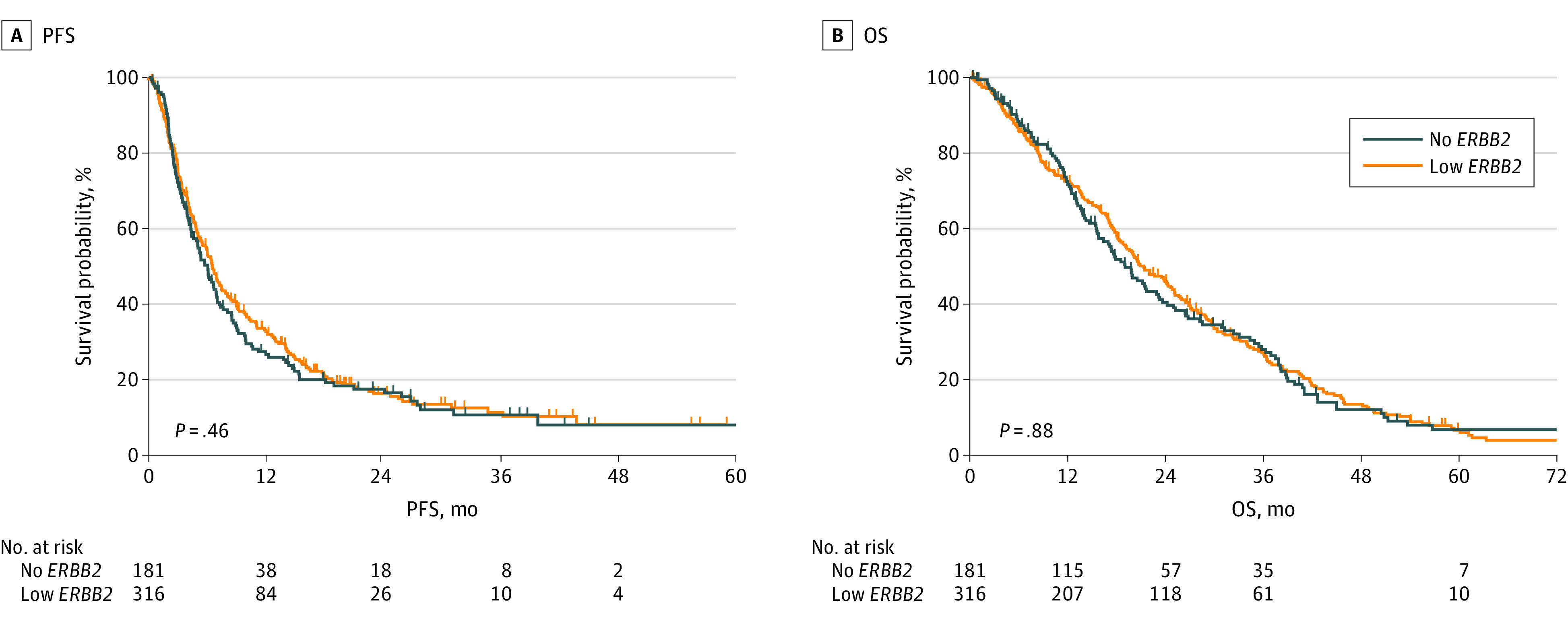
Progression-Free Survival (PFS) and Overall Survival (OS) in Patients With Metastatic Breast Cancer With Hormone Receptor–Positive Low vs No *ERBB2* Expression Treated With Second-line Everolimus or Alpelisib in Combination With Endocrine Therapy

The median follow-up time was 17.5 months with a range from 1 to 110 months. Patients with low vs no *ERBB2* expression treated with first-line CDK4/6is and ET had no statistically significant difference in mPFS (13.0 vs 11.6 months; HR, 1.09; 95% CI, 0.92-1.29; *P* = .27) (Figure 3A) and mOS (32.4 vs 31.2 months; HR, 1.14; 95% CI, 0.92-1.39; *P* = .22) (Figure 3B). In patients with IDC, there was no statistically significant difference in mPFS (12.9 vs 10.1 months; HR, 1.16; 95% CI, 0.95-1.40; *P* = .12) (eFigure 3A in Supplement 1) and mOS (34.2 vs 31.1 months; HR, 1.20; 95% CI, 0.94-1.53; *P* = .13) (eFigure 3B in Supplement 1). In patients with ILC, there was no statistically significant difference in mPFS (12.1 vs 16.6 months; HR, 0.91; 95% CI, 0.60-1.36; *P* = .46) (Figure 4A) and mOS (28.8 vs 34.7 months; HR, 0.95; 95% CI, 0.59-1.52; *P* = .83) (eFigure 4B in Supplement 1).

### Outcomes in Patients Treated With Second-line CDK4/6is, Everolimus, or Alpelisib and ET

The median follow-up time was 12.4 months with a range from 1 to 78 months. When comparing patients with low vs no *ERBB2* expression treated with second-line CDK4/6is and ET, there was no statistically significant difference in mPFS (7.3 vs 7.1 months; HR, 1.20; 95% CI, 0.83-1.72; *P* = .31) (eFigure 5A in Supplement 1) and mOS (31.5 vs 24.9 months; HR, 1.24; 95% CI, 0.80-1.92; *P* = .32) (eFigure 5B in Supplement 1). When comparing patients with low vs no *ERBB2* expression treated in the second-line setting with everolimus or alpelisib in combination with ET, there was no statistically significant difference in mPFS (6.5 vs 6.0 months; HR, 1.08; 95% CI, 0.87-1.33; *P* = .46) (Figure 4A) and mOS (21.3 vs 19.0 months; HR, 1.01; 95% CI, 0.82-1.25; *P* = .88) (Figure 4B).

## Discussion

To our knowledge, this is the largest cohort study to quantify the prognostic impact of HR-positive mBC with low *ERBB2* expression treated with TT and ET and the first to compare outcomes of patients with HR-positive mBC with low *ERBB2* expression across major histologic subtypes (IDC vs ILC). Until now, all prior studies^12,14,15^ had limited numbers of participants and reached different conclusions. Smaller prior retrospective studies disclosed inferior mPFS in patients with HR-positive mBC with low *ERBB2* expression treated with CDK4/6is and ET when compared with patients with HR-positive mBC with no *ERBB2* expression and concluded that low *ERBB2* expression is a potential marker for CDK4/6i efficacy.^12,14^ Our large cohort study refutes those claims given that there were no statistically significant differences in outcomes between the 2 groups. In fact, our study suggests that low *ERBB2* expression status has no prognostic value and is not associated with distinctive clinicopathologic features compared with patients with HR-positive mBC with no *ERBB2* expression treated with TT and ET. Those findings are supportive of previously published data that suggest that low *ERBB2* expression is not associated with CDK4/6i benefit^15,16^ and that it is not a distinct entity in breast cancer by clinical factors assessed so far.^13,17^

One reason for this lack of statistical difference is that the pathologic distinction between no and low *ERBB2* expression is difficult given that the testing methods may be associated with significant variability in *ERBB2* expression^10^ and highly dependent on the reading pathologist.^7^ Additionally, it remains possible that *ERBB2* expression may not be associated with benefit from the newly approved trastuzumab deruxtecan given that the DAISY (Study of DS-8201a, an Antibody Drug Conjugate for Advanced Breast Cancer Patients, With Biomarkers Analysis) phase 2 trial preliminarily showed activity (overall response rate of 30%) with trastuzumab deruxtecan in cases with no *ERBB2* expression.^18^ Given that *ERBB2* is expressed on normal breast cells,^19^ the DAISY trial results might hypothetically suggest that the benefit seen from trastuzumab deruxtecan is irrespective of *ERBB2* expression on breast cancer cells and can be accomplised by trastuzumab deruxtecan binding to *ERBB2 *present on healthy cells and then releasing the chemotherapy moiety (deruxtecan) to the tumor extracellular speace, leading to cancer cell death via the bystander effect. Although the finding was not statistically significant, patients with HR-positive ductal histologic disease had numerically better outcomes in the low *ERBB2* expression group compared with the no *ERBB2* expression group (eFigure 1A and B and eFigure 3A and B in Supplement 1), whereas patients with lobular histologic disease had numerically better outcomes in the no *ERBB2* expression group compared with the low *ERBB2* expression group (eFigure 2A and B and eFigure 4A and B in Supplement 1).

### Limitations

Our study has several limitations, mainly because it is a single-center and observational study. Therefore, the results of this study need further prospective validations to confirm whether low *ERBB2* expression should affect treatment decisions for patients with HR-positive mBC.

## Conclusions

In this cohort study, low *ERBB2* expression was not associated with survival outcomes in patients with HR-positive mBC treated with TT plus ET. The results support the current practice in which low *ERBB2* expression should not influence endocrine-based therapy decision-making.

## Notes

Supplement 1. **eTable 1.** Demographic and Clinicopathological Characteristics of IDC Patients **eTable 2.** Demographic and Clinicopathological Characteristics of ILC Patients **eFigure 1.** Progression-Free Survival (A) and Overall Survival (B) in Patients With Hormone Receptor (HR)-Positive HER2-Low Versus HER2 0 Metastatic Invasive Ductal Carcinoma Treated With Targeted Therapy in Combination With Endocrine Therapy **eFigure 2.** Progression-Free Survival (A) and Overall Survival (B) in Patients With Hormone Receptor (HR)-Positive HER2-Low Versus HER2 0 Metastatic Invasive Lobular Carcinoma Treated With Targeted Therapy in Combination With Endocrine Therapy **eFigure 3.** Progression-Free Survival (A) and Overall Survival (B) in Patients With Hormone Receptor (HR)-Positive HER2-Low Versus HER2 0 Metastatic Invasive Ductal Carcinoma Treated With First-Line Cyclin-Dependent Kinase 4 and 6 Inhibitors in Combination With Endocrine Therapy **eFigure 4.** Progression-Free Survival (A) and Overall Survival (B) in Patients With Hormone Receptor (HR)-Positive HER2-Low Versus HER2 0 Metastatic Invasive Lobular Carcinoma Treated With First-Line Cyclin-Dependent Kinase 4 and 6 Inhibitors in Combination With Endocrine Therapy **eFigure 5.** Progression-Free Survival (A) and Overall Survival (B) in Patients With Hormone Receptor (HR)-Positive HER2-Low Versus HER2 0 Metastatic Breast Cancer Treated With Second-Line Cyclin-Dependent Kinase 4 and 6 Inhibitors in Combination With Endocrine Therapy

Supplement 2.
Data Sharing Statement
