# Supplementary material for: Survival Outcomes in Patients With Hormone Receptor–Positive Metastatic Breast Cancer With Low or No ERBB2 Expression Treated With Targeted Therapies Plus Endocrine Therapy
Source: JAMA Netw Open. 2023 May 11;6(5):e2313017. doi: 10.1001/jamanetworkopen.2023.13017 (PMC10176119; doi:10.1001/jamanetworkopen.2023.13017)
Supplement: Supplement 1. — eTable 1. Demographic and Clinicopathological Characteristics of IDC Patients eTable 2. Demographic and Clinicopathological Characteristics of ILC Patients eFigure 1. Progression-Free Survival (A) and Overall Survival (B) in Patients With Hormone Receptor (HR)-Positive HER2-Low Versus HER2 0 Metastatic Invasive Ductal Carcinoma Treated With Targeted Therapy in Combination With Endocrine Therapy eFigure 2. Progression-Free Survival (A) and Overall Survival (B) in Patients With Hormone Receptor (HR)-Positive HER2-Low Versus HER2 0 Metastatic Invasive Lobular Carcinoma Treated With Targeted Therapy in Combination With Endocrine Therapy eFigure 3. Progression-Free Survival (A) and Overall Survival (B) in Patients With Hormone Receptor (HR)-Positive HER2-Low Versus HER2 0 Metastatic Invasive Ductal Carcinoma Treated With First-Line Cyclin-Dependent Kinase 4 and 6 Inhibitors in Combination With Endocrine Therapy eFigure 4. Progression-Free Survival (A) and Overall Survival (B) in Patients With Hormone Receptor (HR)-Positive HER2-Low Versus HER2 0 Metastatic Invasive Lobular Carcinoma Treated With First-Line Cyclin-Dependent Kinase 4 and 6 Inhibitors in Combination With Endocrine Therapy eFigure 5. Progression-Free Survival (A) and Overall Survival (B) in Patients With Hormone Receptor (HR)-Positive HER2-Low Versus HER2 0 Metastatic Breast Cancer Treated With Second-Line Cyclin-Dependent Kinase 4 and 6 Inhibitors in Combination With Endocrine Therapy [file jamanetwopen-e2313017-s001.pdf]

Supplementary Online Content

Mouabbi JA, Singareeka Raghavendra A, Bassett RL Jr, Hassan A, Tripathy D, Layman RM. Survival outcomes in patients with hormone receptor–positive metastatic breast cancer with low or no *ERBB2* expression treated with targeted therapies plus endocrine therapy. *JAMA Netw Open*. 2023;6(5):e2313017. doi:10.1001/jamanetworkopen.2023.13017

eTable 1. Demographic and Clinicopathological Characteristics of IDC Patients

eTable 2. Demographic and Clinicopathological Characteristics of ILC Patients

eFigure 1. Progression-Free Survival (A) and Overall Survival (B) in Patients With Hormone Receptor (HR)-Positive HER2-Low Versus HER2 0 Metastatic Invasive Ductal Carcinoma Treated With Targeted Therapy in Combination With Endocrine Therapy

eFigure 2. Progression-Free Survival (A) and Overall Survival (B) in Patients With Hormone Receptor (HR)-Positive HER2-Low Versus HER2 0 Metastatic Invasive Lobular Carcinoma Treated With Targeted Therapy in Combination With Endocrine Therapy

eFigure 3. Progression-Free Survival (A) and Overall Survival (B) in Patients With Hormone Receptor (HR)-Positive HER2-Low Versus HER2 0 Metastatic Invasive Ductal Carcinoma Treated With First-Line Cyclin-Dependent Kinase 4 and 6 Inhibitors in Combination With Endocrine Therapy

eFigure 4. Progression-Free Survival (A) and Overall Survival (B) in Patients With Hormone Receptor (HR)-Positive HER2-Low Versus HER2 0 Metastatic Invasive Lobular Carcinoma Treated With First-Line Cyclin-Dependent Kinase 4 and 6 Inhibitors in Combination With Endocrine Therapy

eFigure 5. Progression-Free Survival (A) and Overall Survival (B) in Patients With Hormone Receptor (HR)-Positive HER2-Low Versus HER2 0 Metastatic Breast Cancer Treated With Second-Line Cyclin-Dependent Kinase 4 and 6 Inhibitors in Combination With Endocrine Therapy

This supplementary material has been provided by the authors to give readers additional information about their work.

© 2023 Mouabbi JA et al. *JAMA Network Open.*

eTable 1. Demographic and Clinicopathological Characteristics of IDC Patients

Abbreviations: HER2, human epidermal growth factor receptor 2; IDC, invasive ductal carcinoma; CDK4/6is, cyclin-dependent kinase 4 and 6 inhibitors; 1L, first line; 2L: second line.

**Characteristics – no (%)**	**HER2 0 IDC 475**	**HER2 low IDC 867**	**Unadjusted p-value**
**Age – Median in y. (range)**	49 (92 – 24)	50 (87 – 20)	0.73
**Race**			
**Black**	39 (8.2)	90 (10.3)	0.13
**Hispanic**	50 (10.5)	63 (7.2)	
**White**	346 (72.8)	646 (74.5)	
**Other**	40 (8.5)	68 (8.0)	
**Menopausal Status**			
**Pre**	264 (55.5)	463 (53.4)	0.45
**Post**	211 (44.5)	404 (46.6)	
**Estrogen Receptor**			
**Positive**	449 (94.5)	833 (96.0)	0.21
**Negative**	26 (4.5)	34 (4.0)	
**Progesterone Receptor**			
**Positive**	391 (82.3)	713 (82.2)	0.99
**Negative**	84 (17.7)	154 (17.8)	
**Targeted therapy**			
**CDK4/6i**	320 (67.3)	592 (68.2)	0.91
**1L**	260 (81.5)	494 (83.5)	
**2L**	60 (18.5)	98 (16.5)	
**Everolimus**	148 (31.1)	261 (30.1)	
**Alpelisib**	7 (4.6)	14 (1.7)	

© 2023 Mouabbi JA et al. *JAMA Network Open.*

eTable 2. Demographic and Clinicopathological Characteristics of ILC Patients

Abbreviations: HER2, human epidermal growth factor receptor 2; ILC, invasive lobular carcinoma; CDK4/6is, cyclin-dependent kinase 4 and 6 inhibitors; 1L, first line; 2L: second line.

**Characteristics – no (%)**	**HER2 0 IDC 97**	**HER2 low IDC 146**	**Unadjusted p-value**
**Age – Median in y. (range)**	53 (84 – 34)	55 (81 – 30)	0.77
**Race**			
**Black**	2 (2.0)	9 (6.1)	0.076
**Hispanic**	7 (7.2)	17 (11.6)	
**White**	79 (81.4)	115 (78.7)	
**Other**	9 (9.4)	5 (3.6)	
**Menopausal Status**			
**Pre**	45 (46.3)	56 (38.3)	0.23
**Post**	52 (53.7)	90 (61.7)	
**Estrogen Receptor**			
**Positive**	95 (97.9)	145 (99.3)	0.56
**Negative**	2 (2.1)	1 (0.7)	
**Progesterone Receptor**			
**Positive**	83 (85.5)	117 (80.1)	0.31
**Negative**	14 (14.5)	29 (19.9)	
**Targeted therapy**			
**CDK4/6i**	67 (69.0)	105 (71.9)	0.62
**1L**	65 (97.0)	93 (88.5)	
**2L**	2 (3.0)	12 (11.5)	
**Everolimus**	27 (27.8)	39 (26.7)	
**Alpelisib**	3 (3.2)	2 (1.4)	

© 2023 Mouabbi JA et al. *JAMA Network Open.*

eFigure 1. Progression-Free Survival (A) and Overall Survival (B) in Patients With Hormone Receptor (HR)-Positive HER2-Low Versus HER2 0 Metastatic Invasive Ductal Carcinoma Treated With Targeted Therapy in Combination With Endocrine Therapy

Abbreviations: PFS, progression-free survival; OS, overall survival; HR, hormone receptor; HER2, human epidermal growth factor receptor 2; TT, targeted therapy; ET, endocrine therapy; IDC, invasive ductal carcinoma.

A **Progression-free Survival in HR+ metastatic IDC Treated with TT + ET**

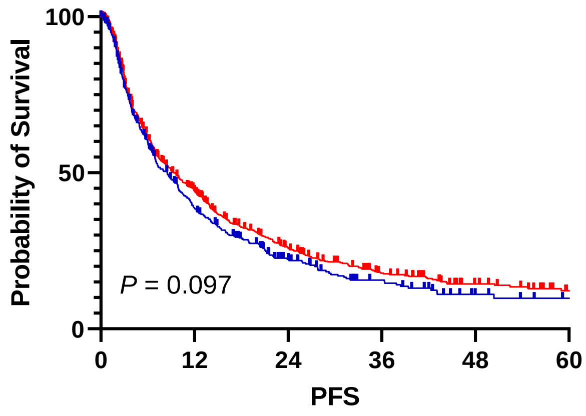

At Risk	0	12	24	36	60
HER2 0	464	149	63	32	6
HER2 low	859	315	135	69	13

B **Overall Survival in HR+ metastatic IDC Treated with TT + ET**

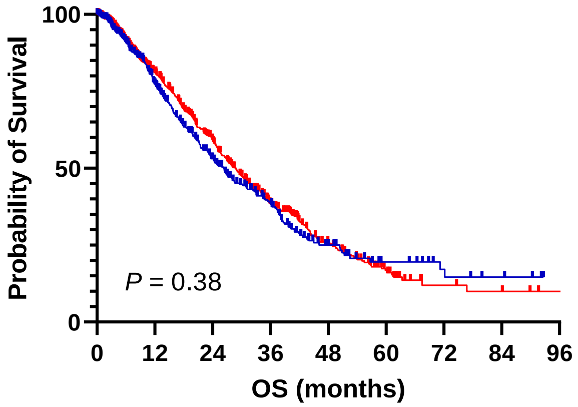

At Risk	0	12	24	36	60
HER2 0	466	301	159	88	14
HER2 low	861	587	341	169	25

© 2023 Mouabbi JA et al. *JAMA Network Open.*

eFigure 2. Progression-Free Survival (A) and Overall Survival (B) in Patients With Hormone Receptor (HR)-Positive HER2-Low Versus HER2 0 Metastatic Invasive Lobular Carcinoma Treated With Targeted Therapy in Combination With Endocrine Therapy

Abbreviations: PFS, progression-free survival; OS, overall survival; HR, hormone receptor; HER2, human epidermal growth factor receptor 2; TT, targeted therapy; ET, endocrine therapy; ILC, invasive lobular carcinoma.

A

Progression-free Survival in HR+ metastatic ILC Treated with TT + ET

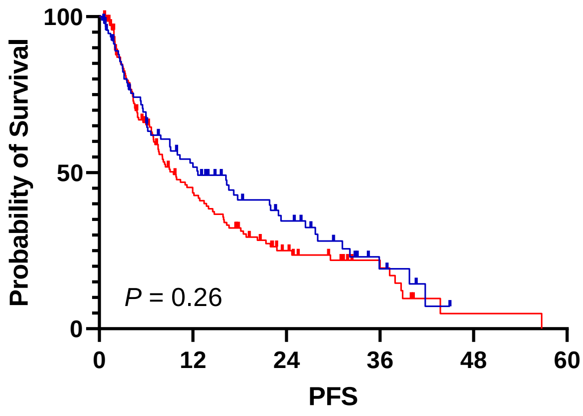

At Risk	0	12	24	36	60
HER2 0	95	41	21	5	1
HER2 low	146	53	20	9	1

B

Overall Survival in HR+ metastatic ILC Treated with TT + ET

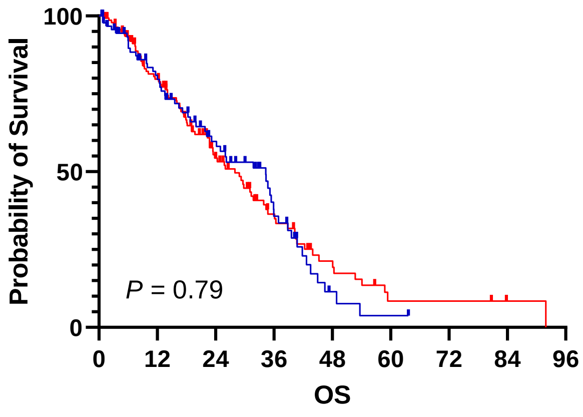

At Risk	0	12	24	36	60
HER2 0	95	64	38	17	1
HER2 low	146	96	49	24	4

© 2023 Mouabbi JA et al. *JAMA Network Open.*

eFigure 3. Progression-Free Survival (A) and Overall Survival (B) in Patients With Hormone Receptor (HR)-Positive HER2-Low Versus HER2 0 Metastatic Invasive Ductal Carcinoma Treated With First-Line Cyclin-Dependent Kinase 4 and 6 Inhibitors In Combination With Endocrine Therapy

Abbreviations: PFS, progression-free survival; OS, overall survival; HR, hormone receptor; HER2, human epidermal growth factor receptor 2; 1L, first line; CDK4/6is, cyclin-dependent kinase 4 and 6 inhibitors; ET, endocrine therapy; IDC, invasive ductal carcinoma.

A **Progression-free Survival in HR+ metastatic IDC Treated with 1L CDK4/6i + ET**

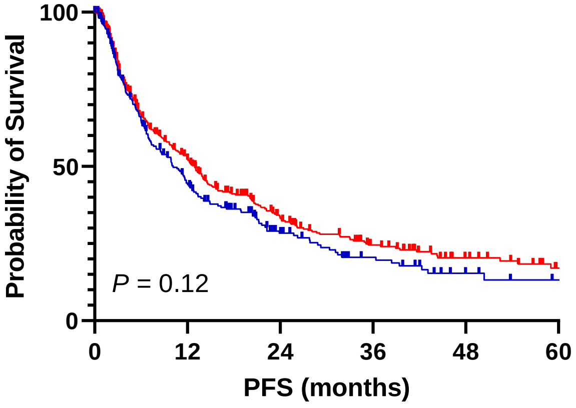

At Risk	0	12	24	36	60
HER2 0	260	96	42	21	5
HER2 low	470	195	92	51	10

B **Overall Survival in HR+ metastatic IDC Treated with 1L CDK4/6i + ET**

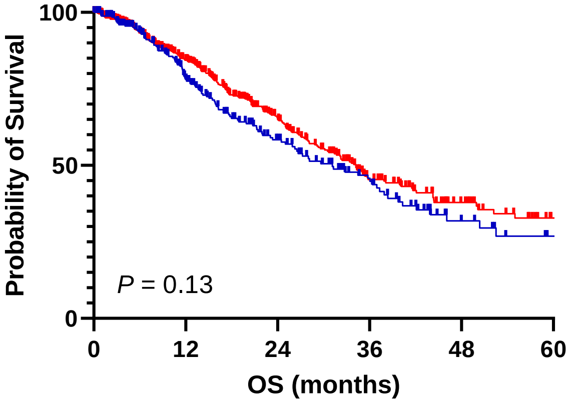

At Risk	0	12	24	36	60
HER2 0	261	162	84	45	8
HER2 low	470	313	117	88	15

© 2023 Mouabbi JA et al. *JAMA Network Open.*

eFigure 4. Progression-Free Survival (A) and Overall Survival (B) in Patients With Hormone Receptor (HR)-Positive HER2-Low Versus HER2 0 Metastatic Invasive Lobular Carcinoma Treated With First-Line Cyclin-Dependent Kinase 4 and 6 Inhibitors in Combination With Endocrine Therapy

Abbreviations: PFS, progression-free survival; OS, overall survival; HR, hormone receptor; HER2, human epidermal growth factor receptor 2; 1L, first line; CDK4/6is, cyclin-dependent kinase 4 and 6 inhibitors; ET, endocrine therapy; ILC, invasive lobular carcinoma.

A **Progression-free Survival in HR+ metastatic ILC Treated with 1L CDK4/6i + ET**

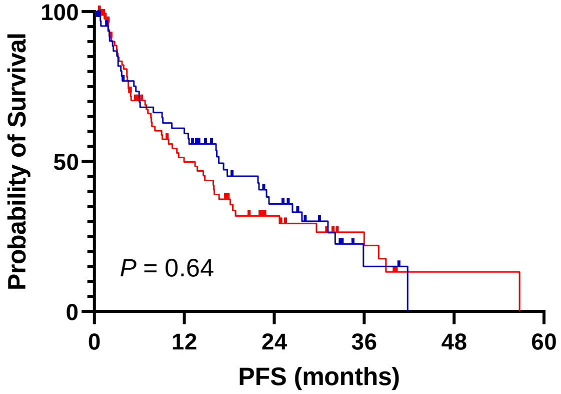

At Risk	0	12	24	36	60
HER2 0	65	34	16	3	1
HER2 low	87	34	14	6	1

B **Overall Survival in HR+ metastatic ILC Treated with 1L CDK4/6i + ET**

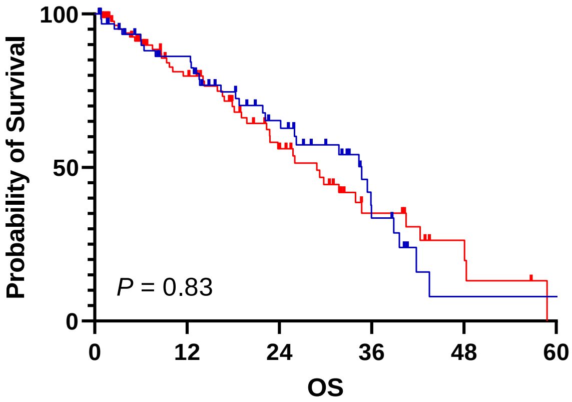

At Risk	0	12	24	36	60
HER2 0	65	47	25	9	1
HER2 low	87	53	27	11	1

© 2023 Mouabbi JA et al. *JAMA Network Open.*

eFigure 5. Progression-Free Survival (A) and Overall Survival (B) in Patients With Hormone Receptor (HR)-Positive HER2-Low Versus HER2 0 Metastatic Breast Cancer Treated With Second-Line Cyclin-Dependent Kinase 4 and 6 Inhibitors in Combination With Endocrine Therapy

Abbreviations: PFS, progression-free survival; OS, overall survival; HR, hormone receptor; HER2, human epidermal growth factor receptor 2; mBC, metastatic breast cancer; 2L, second line; CDK4/6is, cyclin-dependent kinase 4 and 6 inhibitors; ET, endocrine therapy.

A

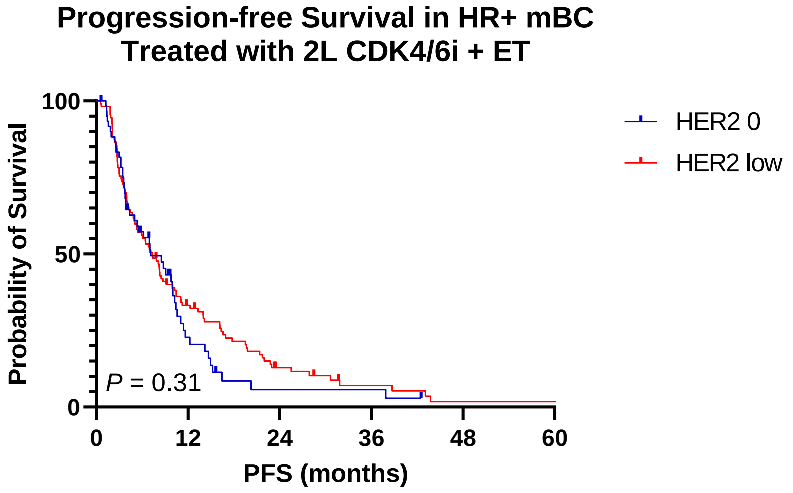

At risk	0	12	24	36	48
HER 0	61	10	3	2	1
HER2 low	110	33	17	5	2

B

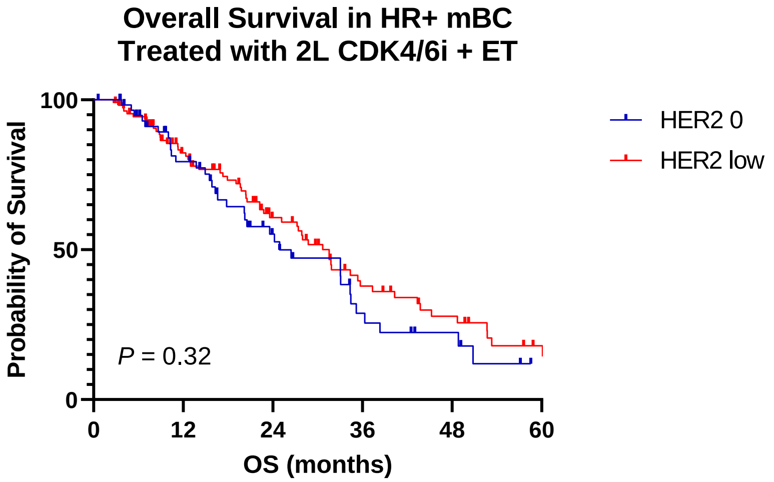

At risk	0	12	24	36	60
HER 0	61	41	21	9	1
HER2 low	110	76	43	22	5

© 2023 Mouabbi JA et al. *JAMA Network Open.*
