# Supplementary material for: Survival Outcomes in Patients With Hormone Receptor–Positive Metastatic Breast Cancer With Low or No ERBB2 Expression Treated With Targeted Therapies Plus Endocrine Therapy
Source: JAMA Netw Open. 2023 May 11;6(5):e2313017. doi: 10.1001/jamanetworkopen.2023.13017 (PMC10176119; doi:10.1001/jamanetworkopen.2023.13017)
Supplement: Supplement 2. — Data Sharing Statement [file jamanetwopen-e2313017-s002.pdf]

Data Sharing Statement

Mouabbi. Survival Outcomes in Patients With Hormone Receptor–Positive Metastatic Breast Cancer With Low or No ERBB2 Expression Treated With Targeted Therapies Plus Endocrine Therapy. *JAMA Netw Open*. Published May 11, 2023.
doi:10.1001/jamanetworkopen.2023.13017

Data
Data available: No

Additional Information
Explanation for why data not available: The data that support the findings of this study are available from the corresponding author, upon reasonable request
